# Noncoding RNA localisation mechanisms in chromatin regulation

**DOI:** 10.1186/1758-907X-3-2

**Published:** 2012-01-31

**Authors:** Aditi Kanhere, Richard G Jenner

**Affiliations:** 1 Division of Infection and Immunity and UCL Cancer Institute, University College London, 72 Huntley Street, London, WC1E 6BT, UK

**Keywords:** noncoding RNA, transcription factor, RNA-protein interaction, chromatin, X inactivation

## Abstract

An important challenge in biology has been to understand how cell-type-specific expression programs are orchestrated through regulated access to chromatin. Knowledge of the interaction between noncoding RNAs (ncRNAs) and chromatin regulators has the potential to help answer such questions, but how ncRNAs target chromatin regulators to specific sites in the genome is not well understood. Recently, Jeon and Lee proposed that DNA-binding proteins act as a bridge between ncRNAs and their target sites in chromatin. In this minireview, we examine their findings and place them in the wider context of how chromatin regulator-RNA complexes are targeted to specific sites in chromatin.

## Introduction

A number of noncoding RNAs (ncRNAs) have been demonstrated to play a role in transcriptional or chromatin regulation through their interaction with chromatin-modifying enzymes and transcription factors. Some of these RNAs can be visualised to be associated with specific loci, most strikingly the coating of the inactive X chromosome (Xi) by X inactive-specific transcript (Xist) RNA. Modulation of a number of ncRNAs leads to changes in the targeting of regulatory complexes to specific genomic sites. Mechanisms must therefore be in operation that direct ncRNA-protein complexes to specific sites in the genome.

### Role of the DNA-binding protein YY1 in the localisation of Xist RNA

**Figure 1:**
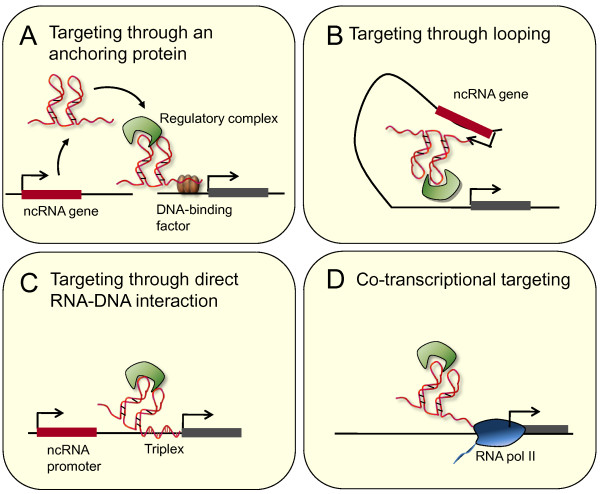
**Potential mechanisms through which chromatin regulator-RNA complexes target specific sites in the genome**. A. Targeting through a sequence-specific DNA binding protein. B. Targeting through looping of the ncRNA locus to the site of activity. C. Targeting through direct RNA-DNA interaction, for example triplex formation. D. RNA as a mechanism to target regulatory proteins to sites of transcription.

Dosage compensation in female mammals is achieved by silencing one copy of the two X chromosomes, termed 'X-chromosome inactivation' (XCI). The long ncRNA Xist is the key factor in initiating this process [1]. Xist is transcribed from the X-inactivation centre (*Xic*) of the future Xi and progressively coats the chromosome. This is accompanied by the appearance of repressive chromatin modifications, including those catalysed by polycomb repressive complex 2 (PRC2). Xist RNA has been proposed to play a direct role in the recruitment of PRC2 through protein-RNA interaction [2]. An unresolved question has been how the Xist-PRC2 complex becomes exclusively localised to the future Xi. Jeon and Lee addressed this problem by studying Xist expression from an inducible transgene in post-XCI embryonic fibroblasts and using *in situ *hybridization and RT-PCR to distinguish between endogenous and transgenic Xist expression [3]. Transgenic Xist RNA localised to the transgene, but, surprisingly, endogenous Xist RNA, normally localised only to Xi, was also seen to migrate to the transgene locus. Seeking to identify a DNA element necessary for this accumulation of Xist RNA, the authors identified a cluster of three Yin Yang 1 (YY1) binding sites within the Xist transgene, and, consistent with this, found that knockdown of YY1 expression also abrogated Xist localisation. Reasoning that YY1 might act as a bridge between the *Xic *and Xist RNA, Jeon and Lee found that Xist and YY1 interact in cells and that YY1 interacts with the Xist repeat C region *in vitro*. Consistent with this finding, an earlier study conducted at the Lee laboratory showed that targeting the C region with locked nucleic acid (LNA) oligonucleotides caused displacement of Xist from the Xi [4]. On the basis of these data, the authors proposed that YY1 acts to anchor the Xist-PRC2 complex to the *Xic *by simultaneously binding to Xist DNA and RNA [3] (Figure 1A).

A number of further experiments could be performed to test the model of Jeon and Lee [3]. Is the Xist C repeat region needed for accumulation of Xist at the *Xic*? Does Xist bind YY1 and PRC2 simultaneously, and are the YY1 DNA-binding sites and the RNA repeat C region needed for PRC2 recruitment to the *Xic*? Can YY1 bind to DNA and RNA simultaneously, and which parts of the protein are required? How does Xist proceed to coat the Xi after initial association with the Xic? The authors showed that YY1 specifically binds to Xist exon1 on the Xi and not on the Xa, which raises the question of what causes this specificity in YY1 binding [3]. It is possible that YY1 does not bind to the nucleation centre on Xa because of the presence of a heterochromatic structure induced by the Xist antisense ncRNA Tsix [5]. YY1 binding sites are also common in the genome, so what prevents Xist accumulation at these positions? Perhaps other factors are involved, such as the RNA-binding nuclear scaffold protein hnRNPU/SP120/SAF-A, which is also required for Xist accumulation on the Xi [6]. Alternatively, in the absence of artificial transgenes, Xist RNA may be captured only by YY1 bound in *cis*.

### Do DNA-binding proteins target other noncoding RNAs to specific sites in chromatin?

The mechanism identified by Jeon and Lee [3] may be responsible for the targeting of other ncRNAs to specific sites in chromatin. The long intergenic RNA Hox antisense intergenic RNA (HOTAIR) is expressed from the *HOXC *locus and modulates histone H3 lysine 27 (H3K27) methylation at *HOXD *and several other sites in the genome in *trans *[7,8]. HOTAIR interacts with PRC2 and the H3K4me2 demethylase complex lysine-specific demethylase 1 (LSD1), thereby bridging two activities which function to promote maintenance of the repressed state [8]. Authors of two recent papers introduced methods termed 'chromatin isolation by RNA purification' (ChIRP) [9] and 'capture hybridization analysis of RNA targets' (CHART) [10], respectively, that use biotinylated oligonucleotides to enrich for DNA sequences associated with a particular RNA. ChIRP was used to isolate HOTAIR-associated chromatin and to identify the enriched DNA sequences by next-generation sequencing [9]. The authors found that HOTAIR commonly nucleates at GA-rich DNA, raising the possibility that the specific targeting of PRC2 and LSD1 by HOTAIR might occur through a YY1-like factor binding to a GA-rich motif.

### Noncoding RNA targeting through chromosomal looping

Another potential mechanism involved in the targeting of ncRNAs to distant DNA target sites is the proximity between loci induced by chromosomal looping (Figure 1B). The long ncRNA HOXA transcript at the distal tip (HOTTIP) is transcribed from the 5' end of the *HOXA *locus and interacts with mixed-lineage leukaemia (MLL) H3K4 methyltransferase complexes through WD repeat-containing protein 5 (WDR5) [11]. Chromosomal conformation capture shows that chromosomal looping brings *HOTTIP *into contact with downstream parts of *HOXA*, and it has been proposed that this allows HOTTIP RNA to target WDR5-MLL to downstream genes [11]. Chromosomal looping may also be important for localisation of enhancer RNAs (eRNAs) to protein-coding genes [12-14]. Knockdown of eRNAs often leads to repression of neighbouring protein-coding genes, suggesting that they play a role in enhancer function [13]. One could imagine that interaction between chromosomal loops may also act to bring ncRNA loci into contact with more distant sites in *trans*, including those on different chromosomes.

### Noncoding RNA targeting by triplex formation with DNA

Direct interactions between RNA and DNA sequences also provide a potential mechanism by which ncRNAs target chromatin regulators to specific sites (Figure 1C). Short promoter RNAs (pRNAs) transcribed from between ribosomal RNA (rRNA) genes directly interact with the nucleolar remodeling complex (NoRC), and this interaction is necessary for association of NoRC with the promoters of rRNA genes in the nucleolus [15]. As for Xist, the regions of the RNA required for NoRC binding and RNA targeting appear to be distinct. A stem-loop structure within the middle of the RNA is necessary for interaction with NoRC [16], but a short sequence towards the 5' end is required for recruitment of the RNA to nucleoli [16,17]. This region corresponds to the DNA element T_0_, and *in vitro *gel shift and protection assays suggest that it forms a triplex structure with the T_0 _DNA sequence. In addition to allowing the RNA to associate with rRNA promoters, this triplex structure is recognised by DNMT3B and is required for DNA methylation [17]. Whether triplex formation occurs between ncRNAs and genomic DNA in cells is unclear, but if it does, it could play a general role in targeting ncRNAs to specific sites.

### Targeting regulatory proteins to sites of noncoding RNA transcription

RNA can target transcriptional regulator proteins in *cis *as it is being transcribed (Figure 1D). The 60-nucleotide transactivation response (TAR) RNA is produced after RNA polymerase II (RNA Pol II) initiation at the HIV long-terminal repeat [18]. In the absence of the HIV protein Tat, further elongation by RNA Pol II is inefficient because of the action of the negative regulatory factors DRB sensitivity-inducing factor (DSIF) and negative elongation factor (NELF) [19]. Tat binds to TAR during transcription and recruits positive transcriptional elongation factor b, or P-TEFb, which then phosphorylates RNA Pol II, DSIF and NELF, resulting in mRNA production [19-22]. A similar mechanism may also function in the targeting of repressive complexes to sites of transcription. Short ncRNAs are transcribed from CpG islands at the 5' end of human genes [23,24]. The RNAs are produced from sites distinct from the gene promoter, and their expression is anticorrelated with gene activity [24]. Production of these short RNAs in the absence of mRNA transcription is associated with targeting of PRC2 in *cis*, and the RNAs directly interact with PRC2 *in vitro *and in cells [24].

### Summary and outlook

Growing evidence suggests that ncRNAs play an important role in chromatin and transcriptional regulation. How these ncRNAs are localised to specific sites in chromatin is not yet clear, but common themes may be emerging. Jeon and Lee's study [3] outlines a mechanism whereby ncRNAs localise to specific loci through interaction with DNA-binding proteins. A role for transcription factors in targeting chromatin regulator-RNA complexes is consistent with their role in initiating changes in epigenetic modifications. It is possible that multiple targeting mechanisms function together. For example, chromosomal looping may provide a high local concentration of ncRNA near a distant target site, with DNA-binding factors then functioning to tether the ncRNA and the associated regulatory complex to more specific locations. Employing genomic methods such as ChIRP or CHART to measure changes in RNA localisation that accompany experimental perturbations will further help to define the mechanisms involved. Recent results indicating that some presumed ncRNAs are associated with ribosomes [25] highlight the importance of determining which RNAs associate with chromatin and which do not. Future work will lead to the derivation of a more robust set of general principles that govern ncRNA localisation and their role in transcriptional and chromatin regulation.

## Abbreviations

RT-PCR: reverse transcriptase polymerase chain reaction.

## Competing interests

The authors declare that they have no competing interests.

## Authors' contributions

AK and RGJ wrote this review together, and both read and approved the final manuscript.
